# Characterization of family goat farms and determination of risk factors associated with the sanitary qualities of raw milk and fresh cheese in three production areas in Mexico

**DOI:** 10.14202/vetworld.2025.927-938

**Published:** 2025-04-23

**Authors:** Israel Daniel Ricardo González, Laura Hernández Andrade, Edith Rojas Anaya, Gary García Espinosa, Susana Elisa Mendoza Elvira

**Affiliations:** 1 Department of Ruminant Medicine and Animal Husbandry, Faculty of Veterinary Medicine and Animal Husbandry, National Autonomous University of Mexico, Mexico City, Mexico; 2 National Center for Disciplinary Research in Animal Health and Safety, National Institute of Forestry, Agricultural and Livestock Research, Mexico City, Mexico; 3 La Campana Experimental Field, North Central Regional Research Center, National Institute of Forestry, Agricultural and Livestock Research, Chihuahua, Mexico; 4 Department of Poultry Medicine and Zootechnics, Faculty of Veterinary Medicine and Animal Husbandry, National Autonomous University of Mexico, Mexico City, Mexico; 5 Virology Laboratory, Faculty of Higher Studies of Cuautitlan, National Autonomous University of Mexico, Cuautitlan Izcalli, Mexico

**Keywords:** family goat farms, fresh cheese, hygienic-sanitary quality, Mexico, raw milk, risk factors

## Abstract

**Background and Aim:**

Family goat farming typically involves small herds managed with minimal infrastructure, leading to products of lower hygienic quality. This study aimed to characterize family goat farms in three distinct regions of Mexico (Durango, Campeche, and Querétaro) and to evaluate hygienic-sanitary indicators and associated risk factors affecting the quality of raw milk and fresh cheese.

**Materials and Methods:**

Seven representative family goat farms were selected based on specific inclusion criteria: Absence of reproductive management, seasonal milk production, manual milking, and artisanal cheese production. Paired samples of bulk raw milk and fresh cheese were collected from each farm. Samples underwent microbiological analyses, including total plate count (TPC), total coliform count (TCC), somatic cell count (SCC), and mold and yeast counts. Surveys addressing animal management, milking, cheese manufacturing, and sales practices were administered. Statistical analyses encompassed descriptive statistics, analysis of variance, cluster analysis, Fisher’s exact tests, and logistic regression.

**Results:**

Among raw milk samples, only two farms met acceptable standards for TPC, SCC, and yeast counts according to Mexican regulations, while none complied for TCC. Similarly, cheese samples from two farms met standards for TPC, yeast, and molds, though none met the standards for TCC. Risk factors significantly associated with poor hygienic quality included inadequate pen hygiene, improper teat cleaning, failure to apply post-dip treatments, deficient hand washing, unsuitable milking techniques, lack of milk pasteurization, and insufficient refrigeration practices. Cluster analysis identified two distinct farm groups differentiated by management practices and hygienic standards, correlating with substantial differences in microbial quality indicators.

**Conclusion:**

The study identified critical gaps in the implementation of good livestock and manufacturing practices among family goat farms in Mexico. Key risk factors contributing to elevated microbial contamination included poor infrastructure, insufficient hygiene during milking and cheese processing, and inadequate storage conditions. The findings emphasize the necessity of promoting standardized hygienic practices and infrastructure improvements to enhance the sanitary quality of milk and cheese products from family goat farming systems.

## INTRODUCTION

In Mexico, goat milk production systems have become polarized, resulting in considerable differences. On the one hand are specialized systems, whose owners have a greater capacity for economic investment, educational level, and acceptance of productive tech-nology; on the other hand, are family systems, which are productive farms made up of small herds managed directly by a shepherd who performs all management activities with help from family. In general, these farms have limited infrastructure, and their productivity levels are very low [1].

In general, family goat farms are small and rustic, with very low milk production levels. This is due to several factors, such as a small number of animals (between 5 and 50), the use of family labor to manage the animals in places close to the producers’ houses in a stable or semistable manner, little or no infrastructure, and the low genetic potential of Creole goats [1–3].

The hygienic-sanitary qualities of raw milk as well as those of cheeses produced in this type of farm are generally poor; health indicators such as the total plate count (TPC) (mesophilic aerobic bacteria), the determination of the total coliform count (TCC), as well as the determination of molds and yeasts can be predictors of the hygienic-sanitary standards in milk, while in the case of cheeses, the total coliform plate count and the determination of molds and yeasts are mainly applied [4, 5].

Despite the substantial role of family goat farming systems in rural economies, there remains limited comprehensive research regarding the specific hygienic-sanitary challenges these systems face, particularly in diverse climatic and regional contexts. Existing studies often focus on commercial dairy farms, neglecting smaller-scale family operations. Consequently, there is a gap in understanding the precise risk factors and management practices influencing the microbiological quality of raw milk and artisanal goat cheeses prod-uced under traditional, family-managed conditions in Mexico.

The present study aims to address this research gap by systematically characterizing family goat farms across three ecologically and culturally distinct regions of Mexico – Durango, Campeche, and Querétaro – and identifying critical risk factors influencing the hygienic-sanitary quality of raw goat milk and fresh artisanal cheese. This research aims to provide actionable insights for improving sanitary standards through targeted inter-ventions and best-practice recommendations tailored specifically for small-scale, family-operated goat farms.

## MATERIALS AND METHODS

### Ethical approval

Ethical approval for animal research was not required.

### Study period and location

This study was carried out from June 5 to 18, 2021, due to restrictions on mobility and in the management of budgetary resources caused by the COVID-19 pandemic, and to try to match lactations in all the herds sampled because the herds were sampled without any type of induction to estrus or reproductive management.

Seven farms representative of the main goat fresh cheese producing areas were selected under the following inclusion criteria: Family production systems where the animals did not have any type of reproductive management (estrus induction), where milk production was seasonal, goat milking was manual (without milking machine), and fresh cheese production was carried out in an artisanal way.

**Figure 1:**
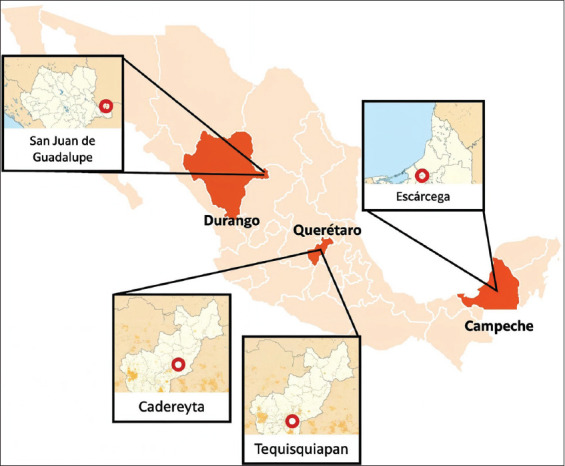
Location of the family goat farms that were used in the study [Source: Image generated with MapChart^©^ 2025 available at: https://www.mapchart.net/).

Two farms in the northern part of the country located in the municipality of San Juan de Guadalupe in the state of Durango were sampled, identified as Durango 01 (D01) and Durango 02 (D02); two in the central area of the country located in the municipality of Cadereyta in the state of Querétaro, identified as Querétaro 01 (Q01) and Querétaro 02 (Q02); and two in the southern zone located in the municipality of Escárcega in the state of Campeche, identified as Campeche 01 (C01) and Campeche 02 (C02). In addition, a farm that has protocols of good livestock and manufacturing practices, identified as DC, located in the municipality of Tequisquiapan in the state of Querétaro, was taken as a reference for subsequent statistical analyses (Figure 1). The characteristics of the regions where the sampling was performed are described in Table 1.

**Table 1:** Characteristics of the sampling sites.

	Cadereyta, Querétaro	Tequisquiapan, Querétaro	San Juan de Guadalupe, Durango	Escárcega, Campeche
Farms sampled	Q01 and Q02	DC	D01 and D02	C01 and C02
Coordinates	20°41′28"N 99°49′08"W	20°31′14"N 99°53′45"W	24°37′52"N 102°46′57"W	18°33′00"N 90°32′00"W
Altitude (masl)	2070	1880	1524	90
Average temperature	12°C−22°C	14°C−20°C	16°C−22°C	26°C−28°C
Precipitation	400−1100 mm	400−600 mm	200−400 mm	1100−1600 mm
Dominant climate	Semi-dry tempered and Sub-humid temperate with rainfall in summer	Tempered semi-dry	Very dry semi-warm	Warm sub-humid with rainfall in summer, moderate humidity
Vegetation	Scrub (51.85%), forest (23.71%), and grassland areas (8.19%) and the jungle (0.54%)	Scrubs (26.29%), forests (6.18%), and jungles (6.13%), Grassland (2.18%) and mezquital (0.06%)	Scrub (80.0%), Grassland (15.2%) and mezquital (1.1%)	Selva (81.41%), other (0.77%), and tular (0.27%)
Agricultural activity	Cultivation of corn, beans, and squash	Corn, beans, wheat, and alfalfa	Corn, alfalfa, sorghum, tomato, chili, and oats	Corn, beans, sorghum, and citrus fruits
Livestock activity	Production of goats, pigs, cattle, meat, and dairy cattle	Production of beef and dairy cattle and goat	Fattening of cattle in pasture and family goat farming	Dairy and beef cattle Sheep and goats

Q01=Querétaro 01, Q02=Querétaro 02, D01=Durango 01, D02=Durango 02, C01=Campeche 01, C02=Campeche 02

### Sampling

In each farm visited, paired samples of bulk raw milk were collected from the milk tank and stored in sterile 50 mL containers under the following specifications: Tube 1 contained no preservatives and was used for the determination of health indicators, while tube 2 contained sodium azide (AppliChem, Germany) at a final concentration of 0.024 g/100 mL as a preservative and was used for a somatic cell count (SCC). Both tubes were transported under refrigerated conditions. In addition, three samples of cheese made from previously sampled raw tank milk were collected. Cheese samples were obtained after the manufacturing and packaging process was completed and were collected in the packaging used by the producer. The samples were preserved and transported in refrigeration (0°C–4°C) to the laboratory for further processing.

### Determination of health indicators and SCCs

The sanitary quality of both the raw milk and cheese was determined in duplicate following the current protocols outlined by Mexican regulations: Preparation of the samples [6], determination of the TPC [7], determination of the total coliforms count (TCC) [8], and determination of molds and yeasts [9]. The SCC was determined using flow cytometry using the Somascope MK2 equipment (NOAK Group, Austria)

### Farm characterization

Surveys were conducted with each of the producers to evaluate the characteristics of the family goat farms and identify risk factors associated with the quality indicators of goat milk and cheese. The surveys consisted of 70 questions related to food and beverage consumption, housing, milking characteristics, milking sites, milking practices, milk management, animal health management, cheese production, and marketing. The Food and Agriculture Organization (FAO) Integrated Agricultural Survey Guide [10], the manual on good livestock practices in the production of goat milk [11], and the FAO Guide to Good Manufacturing Practices in Dairy Products [12] were used as references. The Likert scale was used as follows: 0 = inadequate/not implemented; 1 = adequate or implemented as appropriate. This scale was applied for each question, and the responses were used to obtain the total value for each evaluated item and the general total for each farm.

### Statistical analysis

Descriptive statistics were calculated for the data obtained from the determination of the health indicators of raw milk and fresh cheeses, and analysis of variance and Tukey’s tests were used to identify differences between the sampled regions.

The data obtained from the surveys were analyzed using a hierarchical clustering method to determine the similarities among the respondents in animal management and milking.

Univariate analyses using Fisher’s exact test were performed at a significance level of 95% (p < 0.05) to test associations between independent predictors of questionnaire scores and response variables TPC and SCC for raw milk and TCC for fresh cheeses. The thresholds established for these analyses were as follows: low (<6 Log10 colony-forming unit [CFU]/mL) and high (>6 Log10 CFU/mL) for TPC and low (<6 Log10 cells/mL) and high (>6 Log10 cells/mL) for CSS [12]. For fresh goat cheeses, the following TCC thresholds were used: high (>2.47 Log10 CFU/g) and low (<2.47 Log10 CFU/g). This threshold was chosen because none of the cheeses analyzed presented values that complied with current Mexican regulations, which establish values <100 CFU/g [13]. A logistic regression model was constructed using the predictors identified through univariate analysis. All analyses were performed with IBM Statistical Package for the Social Sciences V25 software (IBM Corp., NY, USA).

## RESULTS

### Cluster survey and analysis

The farms evaluated had a variety of animals (80–200), with an average production of 17.28 kg of cheese per week and an average sale price of $ 92.14 MXN (4.5 USD) (1 MXN = 0.048 USD). The DC and D01 farms had the highest scores, with 60 and 58 points, respectively, out of the 70 points available in the survey, while the remaining farms had scores below 40. Based on the cluster analysis conducted with the data obtained from the surveys, two groups were identified (Table 2). The two clusters were differentiated based on the management practices used for animals and milk and the process used to make fresh cheese. The first group included DC and D01 farms, and the second group included the remaining farms. Importantly, a marked difference was observed between health indicators and SCC (Table 2).

**Table 2:** Scores for milk and fresh goat cheese from family farms.

Location	No. of questions	DC	D01	D02	Q01	Q01	C01	C02
					
		Center	North	North	Center	Center	South	South
Number animals	1 (N/I)	150	180	200	80	126	70	100
Food and beverage consumption	10	9	9	5	3	6	3	4
Accommodations	9	8	8	5	3	4	4	4
Milking characteristics	11	8	8	6	4	6	4	5
Milking management	12	8	7	4	5	6	5	4
Milk handling	6	4	4	3	3	3	3	2
The cheese-making process	17	21	20	16	16	14	17	12
Marketing and sale	5	2	2	1	2	1	1	1
Sales volume (kg/week)	1	28	25	20	12	18	7	11
Sale price ($ NMX/kg)	1 (N/I)	125	110	85	100	90	65	70
Total score	1 (N/I)	60	58	40	36	40	37	32
Cluster		1	1	2	2	2	2	2

N/I=Question not included in subsequent analysis. Q01=Querétaro 01, Q02=Querétaro 02, D01=Durango 01, D02=Durango 02, C01=Campeche 01, C02=Campeche 02

### Health indicators of bulk raw milk

Farms D01 and DC (farm used as a reference) (28.57% of farms evaluated) presented TPC values below the values stipulated by current Mexican regulations and significantly lower than those of the rest of the farms evaluated in the samples analyzed; 100% of the farms evaluated presented TCC values that exceeded the reference value (2 Log10 CFU/mL); however, the mean values of the DC and D01 farms were significantly lower than those of the rest of the farms evaluated. In the case of molds, 100% of the farms remained within the allowable range (up to 2 Log10 CFU/mL), whereas for yeasts, only the D01 and DC farms (28.57% of farms evaluated) remained within the allowable range (up to 2 Log10 CFU/mL). The SCC on only DC, Q02, and D01 farms (42.85% of farms evaluated) remained within the recommended range (up to 1,000,000 cells/mL) (Table 3).

**Table 3:** Average values of health indicators and somatic cell counts found in raw tank milk from family goat farms.

Farm	Total plate count (Log10 CFU/mL)	Total coliform count (Log10 CFU/mL)	Molds (Log10 CFU/mL)	Yeasts (Log10 CFU/mL)	Somatic cell count (Cel/ml)
DC	5.47^b^	3.15^b^	0.48^b^	1.08^b^	524,000
Q01	6.38^a^	5.17^a^	Neg^b^	4.41^a^	1,048,000
Q02	6.32^a^	5.74^a^	Neg^b^	4.56^b^	642,000
D01	5.44^b^	3.09^b^	0.30^b^	1.08^b^	824,000
D02	6.29^a^	4.87^c^	0.00^b^	4.51^a^	1,076,000
C01	6.30^a^	5.32^a^	1.00^a^	4.45^a^	1,248,000
C02	6.49^a^	5.39^a^	1.48^a^	4.97^a^	1,804,000
Reference value	Up to 6	Up to 2	Up to 2	Up to 2	Up to 1,000,000

Values in the same column that do not share the same subscript are significantly different at p *<* 0.05 level on the basis of a two-sided test of equality for column means. Q01=Querétaro 01, Q02=Querétaro 02, D01=Durango 01, D02=Durango 02, C01=Campeche 01, C02=Campeche 02, CFU=Colony-forming unit

### Health indicators of fresh goat cheese

In five of the seven farms evaluated TPC in raw milk before the cheese-making process exceeded 6.07 Log10 CFU/mL, indicating that these samples complied with current national regulations (NMX-F-728-COFOCALEC-2017.) [13, 14]. However, none of the farms evaluated in this study adhered to the international recommended limits for raw milk of 5 Log10 CFU/mL (Food and Drug Administration [FDA]) [15] and 4.69 Log10 CFU/mL (European regula-tions [Standard 92/46/EECC]).

Only the D01 and DC farms (28.57% of farms evaluated) had TPC levels below the levels recommended in a similar study by Renye *et al*. [16] (up to 6 Log10 CFU/mL), none of the farms had TCC levels within the recommended levels (2 Log10 CFU/mL). The DC and D01 farms maintained statistically lower values for these two indicators than the other farms. For molds, DC and D01 farms (28.57% of farms evaluated) remained below the recommended range (up to 2 Log10 CFU/mL), whereas for yeasts, 4 farms (57.14% of farms evaluated) remained below the reference value of up to 2 Log10 CFU/mL (Table 4) [16].

**Table 4:** Average values of health indicators in fresh goat cheese from family goat farms.

Farm	Total plate count (Log10 CFU/mL)	Total coliform count (Log10 CFU/mL)	Molds (Log10 CFU/mL)	Yeasts (Log10 CFU/mL)
DC	3.63^b^	2.30^c^	1.00^a^	Neg^b^
Q01	6.75^a^	4.70^a^	4.55^a^	1.08^b^
Q02	6.69^a^	4.57^a^	4.13^a^	1.73^b^
D01	3.86^b^	2.27^c^	2.42^b^	Neg^b^
D02	6.30^a^	3.78^b^	3.99^b^	3.15^a^
C01	6.47^a^	4.86^a^	4.34^a^	4.04^a^
C02	6.94^a^	4.95^a^	3.66^a^	3.45^a^
Reference value	6.00	2.00	2.70	2.70^a^

Values in the same column that do not share the same subscript are significantly different at the p *<* 0.05 level on the basis of a two-sided test of equality for column means. *Reference values were taken from Renye *et al*. [16]. Q01=Querétaro 01, Q02=Querétaro 02, D01=Durango 01, D02=Durango 02, C01=Campeche 01, C02=Campeche 02, CFU=Colony-forming unit

### Risk factors associated with TPC and SCCs in raw goat milk

The risk factors associated with an increase in TPC were those variables linked to the general characteristics of the herd and the production systems, such as insufficient space for feeders and drinkers (odds ratio [OR] = 5.505), not having a specific place for milking (OR = 3.892), not having a covered milking area (OR = 2.104), improper hand washing before milking (OR = 5.275), improper drying of the teats (OR = 5.275), and improper cooling of milk before the cheese making process (OR = 1.333).

In addition, the following risk factors were associated with an increase in both TPC and CSS: poor hygiene in pens (TPC: OR = 5.275; SCC: OR = 2,000), improper hand washing before milking (TPC: OR = 1,667; SCC: OR = 1.050), improper nipple washing (TPC: OR = 1.667; SCC: OR = 1.050), inadequate milking tech-nique (TPC: OR = 4.276; SCC: OR = 1.467), and use of a post-dip (TPC: OR = 4.276; and SCC: OR = 1.467) (Table 5).

**Table 5:** Risk factors associated with aerobic mesophilic and somatic cell counts in cheese produced by family goat farms in Mexico.

Independent variable	Category	TPC		SCC	
	
		Odds ratio (95% confidence interval)	p-value	Odds ratio (95% confidence interval)	p-value
Feeder and drinker spaces	0=Inadequate 1=Adequate	5.505 (1.687–4.695)	0.048	N/A	N/A
General cleaning of the pens	0=Inadequate 1=Adequate	3.905 (1.978–6.295)	0.000	1.050 (0.023–11.080)	0.000
Specific place for milking	0=No 1=Yes	3.892 (1.695–7.284)	0.048	N/A	N/A
Roofed site with paved floor	0=No 1=Yes	2.104 (1.020–4.434)	0.000	N/A	N/A
Handwashing with soap and water	0=No 1=Yes	5.275 (0.664–3.772)	0.048	2.000 (0.751–5.329)	0.042
Nipple washing with drinking water	0=No 1=Yes	1.667 (0.815–3.409)	0.000	1.050 (0.023–11.080)	0.000
Proper drying of the nipples	0=No 1=Yes	5.275 (0.664–6.772)	0.048	N/A	N/A
Pre-dip use	0=No 1=Yes	5.275 (0.664–6.772)	0.048	N/A	N/A
Proper milking technique	0=No 1=Yes	4.276 (0.067–6.652)	0.000	1.467 (0.067–1.652)	0.000
Post-dip use	0=No 1=Yes	4.276 (0.067–6.652)	0.000	1.467 (0.067–1.652)	0.000
Milk refrigeration	0=No 1=Yes	1.333 (0.757–2.348)	0.059	N/A	N/A

TPC=Total plate count, SCC=Somatic cell count

### Risk factors associated with TCC in fresh goat cheese

Risk factors associated with high TCC included the following variables: insufficient space in feeders and drinkers (OR = 5.505), lack of a specific place for milking (OR = 3.892), improper hand washing before milking (OR = 5.275), improper drying of teats (OR = 5.275), lack of use of sealant (OR = 2.104), storage of milk in dirty containers (OR = 3.892), production of cheese in a place protected from environmental conditions (OR = 3.552), containers without dust protection (OR = 3.892), and lack of refrigeration of the cheese before sale (OR = 5.275). Factors associated with high significance (p = 0.000) were also identified; poor hygiene, pens with a high presence of manure (OR = 3.905), lack of roof and paved floor (OR = 3.892), improper washing of teats (OR = 1.667), lack of use of sealant (OR = 2.104), days of milk storage before processing (OR = 1.667), the lack of pasteurization of milk (OR = 1.667), and poor refrigeration of the finished product (OR = 1.667) (Table 6).

**Table 6:** Risk factors associated with high coliform counts in cheese produced by family goat farms.

Independent variable	Category	TCC	

		Odds ratio Independent variable (0/1) and 95% confidence interval	p-value
Feeder and waterer space	0=Inadequate 1=Adequate	5.505 (1.687–4.695)	0.048
General cleaning of the pens	0=Inadequate 1=Adequate	3.905 (1.978–6.295)	0.000
Specific place for milking	0=No 1=Yes	3.892 (1.695–7.284)	0.048
Roofed site with paved floor	0=No 1=Yes	2.104 (1.020–4.434)	0.000
Handwashing with soap and water	0=No 1=Yes	5.275 (0.664–3.772)	0.048
Nipple washing with drinking water	0=No 1=Yes	1.667 (0.815–3.409)	0.000
Proper drying of nipples	0=No 1=Yes	5.275 (0.664–6.772)	0.048
Pre-dip use	0=No 1=Yes	5.275 (0.664–6.772)	0.048
Post-dip use	0=No 1=Yes	2.104 (1.020–4.434)	0.000
Average number of days of milk storage	0=More than 5 1=Less than 5	1.667 (0.815–3.409)	0.000
Protected area from environmental conditions for cheese production	0=No 1=Yes	3.552 (0.880–8.256)	0.043
Containers protected from dust	0=No 1=Yes	3.892 (1.695–7.284)	0.048
Milk pasteurization	0=No 1=Yes	1.667 (0.815–3.409)	0.000
Finished product allowed to cool	0=No 1=Yes	1.667 (0.815–3.409)	0.000
Refrigeration before sale	0=No 1=Yes	5.275 (0.664–3.772)	0.048

### Logistic regression model

Finally, the logistic regression model indicated that the variables that influenced TPC levels in raw goat’s milk (p < 0.05) dirty pens, use of hand milking, use of non-potable water in teat washing, and lack of use of post-dip; the variables that influenced the level of SCC were: dirty pens, absence or poor hand washing, use of non-potable water in teat washing, inadequate drying of teats, inadequate milking technique, and lack of use of post-dip; and the variables that influenced the level of TCC in the cheese were as follows: lack of use of post-dip, use of unpasteurized milk, and poor cooling of the finished product (Table 7).

**Table 7:** Results of a logistic regression model that included factors significantly associated with TPC, SCC, and TCC in milk and fresh goat cheese.

Variable	β Coefficient	Standard error of β coefficient	Odds ratio	Confidence interval (95%)
TPC				
General cleaning of the pens	2.917	0.088	0.255	0.118–1.458
Milking type	1.120	0.290	0.440	0.202–0.978
Washing of the nipples with drinking water	1.120	0.290	0.440	0.202–0.978
Use of post-dip	2.917	0.088	0.255	0.118–1.458
Constant	0.916	0.837		
SCC				
General cleaning of the pens	0.194	0.659	4.637	1.324–5.344
Handwashing with soap and water	2.100	0.147	0.256	1.432–3.651
Washing of the nipples with drinking water	2.100	0.147	0.256	1.432–3.651
Proper drying of the nipples	0.875	0.350	0.471	0.173–0.938
Use of the proper milking technique	2.100	0.147	1.924	0.641–4.648
Use of post-dip	1.556	0.212	0.855	0.118–0.705
Constant	0.288	0.764		
TCC in cheese				
Use of post-dip	2.917	0.088	4.626	1.532–8.55
Milk pasteurization	2.917	0.088	4.626	1.532–8.55
Finished product allowed to cool	0.467	0.495	0.450	0.128–0.253
Constant	0.916	0.837		

TPC=Total plate count, SCC=Somatic cell count, TCC=Total coliform content

## DISCUSSION

The high TPC levels observed in this study indicate that producers continue to experience various problems related to inadequate hygiene practices; these counts are consistent with previous results by Isidro-Requejo *et al*. [4] and Díaz-Rivera *et al*. [17], from studies carried out in Mexico, with more than 50% of the goat family farms having high TPC levels, coinciding with what was described by Tajonar *et al*. [3], where characteristics such as no or little use of technology and little preventive medicine and poor feeding negatively affect both the health of the herd and the quality of the products obtained.

On the other hand, milk storage conditions in conjunction with the prevailing environmental conditions experienced by herds could favor bacterial growth, especially if late cooling or suboptimal storage temperatures are present; therefore, inadequate temperatures in milk cooling tanks or a lack of optimal environmental conditions may be responsible for high TPC counts [3, 4, 18, 19]. This situation is not exclusive to Mexico; studies carried out in goat farms in Brazil have reported values of up to 7.89 Log10 CFU/mL in raw milk [20, 21]; in Serbia they have reported values of up to 6.69 log10 CFU/mL [21]; and in Sudan with values of up to 6.53 Log10 CFU/mL [22]. These four studies describe the main causes of these high count conditions, such as storage temperature and hygiene of the process of obtaining a milk. The TPC can serve as an indicator not only of the hygiene practices used on farms but also of the suitability of milk stored in tanks for pasteurization and by-product production, with the critical limit being 2.47 Log10 CFU/mL [23].

The pattern for TCC was very similar to that of TPC. The high number of coliforms in raw goat’s milk found in this study coincides with a report from Ethiopia, where the microbiological quality of raw goat’s milk from family goat farms was evaluated and values of 4.17 Log10 CFU/mL were found [24] were found, and with observations from similar studies carried out in Mexico, Brazil, Argentina, and Poland where values of up to 7.8x10^6^ CFU/mL were reported for this indicator [20, 21, 25–27]. The coliform count has been used as an indicator of fecal contamination in water and food; however, it is not strictly related to fecal contamination, and this indicator is known to be associated with high loads of coliform bacteria in the environment and not only in the water used for processing [23, 28]. Although in Mexico, there is no legislation on the presence of this indicator in raw goat’s milk, it has been reported that counts above 2 Log10 CFU/mL are related to hygiene failures during milking and storage [28, 29].

In Mexico, there is no specific regulation for SCC in raw goat’s milk; however, in various studies by Isidro-Requejo *et al*. [4], Food and Drug Administration [15], Díaz-Rivera *et al*. [17], Nuhriawangsa *et al*. [18], Pereira *et al*. [20], and De Siqueira *et al*. [21], the (NMX-F-728-COFOCALEC-2017) standard was usually used, which establishes a limit of up to 1 × 10^6^ cells/mL. Notably, it should be noted that this regulation is focused on dairy cattle; however, there are stricter regulations, such as the one proposed by the FDA, which states that raw milk intended for pasteurization should not exceed 7.5 × 10^5^ cells/mL [15]. The maximum value of SCC found in the present study was 1.8 × 10^6^ cells/mL, which was higher than that reported in similar studies carried out in Serbia, Italy, and the Czech Republic, where the maximum value reported was 1,450,000 cells/mL, these reported values were positively related to the high content of sanitary indicators and poor hygiene conditions in the pen and during milking [30–32]. On the other hand, studies conducted in Latin America have reported levels of up to 1.9 × 10^6^ cells/mL [33]. Importantly, the establishment of standards for SCC in goats’ milk has been controversial due to the lack of consensus on a threshold that can accurately differentiate animals with and without subclinical mastitis [34]. The importance of determining SCC in raw goat’s milk extends beyond predicting mammary gland health in these animals. Koop *et al*. [35] reported a positive relationship between high SCC in milk and health indicators such as TPC and TCC and low sensory quality in cheeses (more than 1.5 × 10^6^ cells/mL) [36], the fat/protein ratio [30, 34], and an increase in the relative concentrations of these microorganisms due to a decrease in production [37].

The TPC values were high for fresh cheeses that do not use a starter culture [28, 38]. These values are similar to those reported for similar studies conducted in Mexico, with values of 6 Log10 CFU/g [25, 39]; in countries with similar manufacturing methods, such as Algeria, with values of 8.88 Log10 CFU/g [40]; in the Czech Republic, with values of 6.62 Log10 CFU/g [41]; in Poland, with values of 3.69 Log 10 CFU/g [27]; and in Serbia, with values of 3.54 Log10 CFU/g [32]. The differences in the values were attributed to the hygienic conditions during the cheese-making process. The differences in the values found can be attributed to the hygienic conditions during the cheese-making process and the lack of a place protected from the weather to produce the cheese. In Mexico, there is no legislation that specifies the maximum allowable amount of TPC in fresh cheeses; however, Renye *et al*. [16] proposed an upper limit of 6 Log10 CFU/g because high levels of this parameter may indicate inadequate storage (inadequate time and/or temperature). Among TPC, there may be pathogenic microorganisms (bacteria that cause foodborne illness) can occur, and many of these microorganisms can modify the organoleptic properties of food [42].

The limit established by Mexican regulations for TC in cheeses made from fresh milk [43] is 2 Log10 CFU/g. None of the cheeses sampled in this study presented values within the specified range. These results are consistent with those reported for studies conducted in the state of San Luis Potosí, Mexico, with values of up to 10.11 Log10 CFU/g [44], and in Oaxaca, Mexico, with values of up to 8.10 Log10 CFU/g [38]. On the other hand, studies evaluating the hygienic quality of fresh organic cheeses in the Czech Republic have reported values of up to 6.73 Log 10 CFU/g [41], whereas values of up to 6.25 Log 10 CFU/g have been reported in Slovakia [45]. Praça *et al*. [46] revealed that high levels of TCC were attributed to poor hygiene at the time of processing and the type and place where the cheeses were made (without protection of environmental conditions and difficult to clean) and may represent the presence of pathogens that cause foodborne diseases.

With respect to mold counts, farms DC, Q01, Q02, and D01 remained within the range stipulated by Mexican regulations [42]. For yeasts, only DC and D01 farms remained within the established range (500 CFU/mL); the remaining family goat farms pres-ented maximum values of 2.69 Log10 CFU/g for yeasts and 14 Log10 CFU/g for molds. These values are similar to those reported in a study conducted in Oaxaca, Mexico, with values of up to 4.82 Log 10 CFU/g [38], and lower than those reported in Venezuela for fresh goat cheese in family goat farms, with values of up to 6.49 Log 10 CFU/g [47]. The presence of molds and yeasts in fresh cheese is mainly associated with the work environment as well as poor hygienic conditions of the equipment, utensils, and storage [38]. On the other hand, yeasts can cause alterations in cheeses, with the most common defects being the taste of mold, changes in texture, excessive gas formation (swelling of cheeses), increased acidity due to stimulation of lactic acid bacteria, discoloration, and surface growth [48].

The cluster analysis revealed the formation of two groups: one that included 28.57% of the sampled farms in which many of the precepts of good livestock practices were applied and another that included 71.43% of the herds evaluated and in which there were serious deficiencies in animal management and health. Poor hygienic management during the collection and storage of raw milk and during the production of fresh cheeses reflects the current situation of family herds in Mexico, demonstrated in studies of family goat farms in San Luis Potosí and Puebla state. [2, 3, 25].

Univariate analysis was used to identify variables associated with TPC and SCC in raw milk; these variables included inadequate milking locations (OR = 3.892), lack of post-dip (OR = 4.276), and factors with greater impact (OR 1.667–4.276), such as poor pen cleanliness, type of milking (use of hand milking), milking place (dirt floor and no roof), inadequate teat washing, poor milking technique, and lack of use of post-dip. In addition, the average days of milk storage (more than five) and the type of feeding were important variables for the count of TPC and SCC, respectively, which is consistent with previously published information on the impact that good livestock practices have on the quality parameters of animal products, specifically raw goat’s milk [4, 18, 33, 49, 50].

In addition to the aforementioned factors, the sanitary quality of fresh cheese was affected by milking practices, the handling of raw milk, and factors associated with the manufacturing process, such as feeder and waterer space (OR = 5,505), the presence of harmful fauna (OR = 2,893), the use of a specific location for milking (OR = 3,892), hand washing with soap and water before milking (OR = 1,667), drying of the teats (OR = 5,275), the use of a post dip (OR = 1,104), cheese production in a place protected from environmental conditions (OR = 3,552), and refrigeration before sale (OR = 5,275). Factors associated with OR values of up to 5,275 were also identified; these factors included inadequate cleaning of pens, use of hand milking, dirt floor and no roof, inadequate teat washing, incorrect milking technique, lack of post-dip use, lack of pasteuri-zation of the milk, and deficient refrigeration of the finished product. These results are very similar to those reported in other studies [24, 33, 50–52], in which poor hygiene practices during the goat rearing and cheese manufacturing process (dirty udders due to lack of udder washing before milking, dirty hands, poor personal hygiene, lack of hygiene at the milking site, and lack of hygiene in the cheese factory) are more likely to increase health indicators or cause foodborne illnesses, in addition to being a limitation for the marketing of cheeses, and are consistent with the recommendations of the manual on good livestock practices for goat milk production published in Mexico [4, 53].

According to the developed logistic regression model, one of the variables that influence novice TPC stops is milking type. In this case, hand-milking incre-ases the risk of contamination if adequate hygienic practices are not followed. According to studies, manual handling under unhygienic conditions can introduce environmental microorganisms, resulting in an increase in the bacterial load in milk. This has been demonstrated in studies carried out in Malaysia where the hygienic quality of raw milk from farms under different handling conditions was evaluated, finding that farms with hand-milking and with poor hygiene in the washing of milkers’ hands presented high levels of TPC [53]. In Mexico, a large part of goat family farms practice hand-milking [3], studies carried out in “La laguna,” Mexico relate hand-milking to high levels of bacterial load [4]. On the other hand, in Indonesia, they also attributed hand-milking to an increase in TCP accounts [18].

It was found that there are variables common to TCP and SCC in the raw milk from the tank evaluated, such as the degree of cleanliness of the pens, since the presence of organic matter increases the possibility of bacterial contamination of the mammary gland [10]. This has been demonstrated by Lopes *et al*. [33], who were able to determine this variable as a factor of resistance. In Mexico, studies characterizing goat farms have shown that most goat family farms do not have adequate facilities or specific cleaning routines in pens, which negatively affects the quality of the raw milk they produce [3].

Milking-related variables were also common to TCP and SCC in the obtained logistic regression model. Variables such as incorrect hand washing, incorrect teat washing, lack of use of post-dip, and incorrect milking routine. These variables can significantly impact novices in TCP and SCC. Milkers who do not wash their hands properly can transfer bacteria to the milking process, thereby increasing microbial contamination. In addition, poor milking techniques can cause stress in goats and allow pathogens to enter the milk. The absence of a post-dip, which acts as a protective barrier against contamination, can result in an increase in bacterial load, which not only affects milk quality [10, 39]. These variables have also been associated with the increase in TCP and SCC studies conducted in different countries, such as Italy [30], Mexico [17,24, 29], Czech Republic [31], Ukraine [19], and Brazil [20, 54], agree that the general milking routine plays a critical role in the hygienic-sanitary quality of raw milk. On the other hand, Lopes *et al*. [33] also associated these variables with the increase in TCP and SCC.

Variables such as the lack of pasteurization of milk and refrigeration of the finished product were variables that were associated with the increase in TCC. Pasteurization is a crucial process that eliminates pathogens and reduces the microbial load, helping to ensure food safety. Without this treatment, raw milk can contain harmful bacteria, including fecal coliforms, which can proliferate during cheese production. In addition, the lack of refrigeration allows these bacteria to multiply even more, increasing the risk of contamination and potential outbreaks of foodborne diseases [10, 39, 40]. In Mexico, a large part of the family cheese factories has poor hygiene conditions as well as sufficient storage conditions for milk (refrigeration) and do not usually pasteurize the milk to produce fresh cheeses [3]. Studies evaluating the quality of fresh cheeses of goat origin conducted in Mexico concluded that one of the causes of high TCC levels is the lack of refrigeration and pasteurization of milk [25].

## CONCLUSION

This study characterized family-operated goat farms across three ecologically diverse regions in Mexico and identified significant hygienic-sanitary deficiencies affecting the microbiological quality of raw goat milk and fresh artisanal cheese. The results highlighted critical risk factors including inadequate hygiene pra-ctices in animal pens, improper teat washing and drying, absence of post-milking teat disinfection (post-dip), substandard handwashing protocols, unsuitable milking techniques, absence of milk pasteurization, and inadequate refrigeration. Notably, only two out of seven evaluated farms met Mexican sanitary regulations for microbial indicators (TPC, SCC, molds, and yeasts), while none complied with established guidelines for TCC.

The comprehensive, multi-regional approach allowed an extensive evaluation of diverse family goat farming systems, providing robust, comparative insights. Methodologically rigorous analyses, including cluster analysis and logistic regression modeling, enhanced the reliability and applicability of the findings, clearly demonstrating the link between management practices and product quality.

A principal limitation was the relatively small sample size (seven farms), potentially restricting the generalizability of the results to all family-operated goat farms across Mexico. Moreover, the cross-sectional nature of the study limits causal inferences, and sea-sonal variations, potentially influencing milk and cheese quality, were not explored extensively.

Future research should incorporate larger sample sizes and longitudinal designs to better capture seasonal effects and establish clearer causal relationships between management practices and hygienic-sanitary outcomes. In addition, investigations examining inter-vention efficacy – such as implementation of good livestock practices, hygiene education programs, or infrastructure improvements – could provide practical insights into improving product safety and quality standards in family-managed goat farming systems. Finally, exploring the impact of identified hygienic-sanitary deficiencies on consumer health and product marketability represents a crucial direction for sub-sequent studies.

## AUTHORS’ CONTRIBUTIONS

IDRG: Sampling and supervision. IDRG and LHA: Laboratory analysis. IDRG, ERA, GGE, and SEME: Data analysis and study design. IDRG: Statistical analysis. IDRG, LHA, ERA, GGE, and SEME: Drafted and revised the manuscript. All authors have read and approved the final manuscript.
